# Inflammation, Statin Pleiotropy, and Cardiovascular Prevention in Women: Established Evidence, Biological Plausibility, and Knowledge Gaps

**DOI:** 10.3390/jcdd13080389

**Published:** 2026-08-14

**Authors:** Rogerio Castellan de Moraes, Paulo Magno Dourado, Viviane Zorzanelli Rocha, Heno Ferreira Lopes, Fernanda Marciano Consolim-Colombo

**Affiliations:** 1 Postgraduate Department, Universidade Nove de Julho (UNINOVE), São Paulo 01504-001, Brazil; rogerio77.rm@gmail.com (R.C.d.M.); fermccolombo@gmail.com (F.M.C.-C.); 2 Hypertension Unit, Heart Institute of Medical School, University of São Paulo, São Paulo 05403-900, Brazil; pmdourado@terra.com.br (P.M.D.); vzrocha@hotmail.com (V.Z.R.)

**Keywords:** cardiovascular disease, women, statins, inflammation, pleiotropic effects, sex differences, lipid lowering, precision medicine

## Abstract

Cardiovascular disease remains the leading cause of death among women, yet female cardiovascular risk continues to be underrecognized, and women remain underrepresented in many cardiovascular trials. Sex-related differences in hormonal exposure, immune regulation, vascular function, pregnancy-related conditions, and clinical presentation contribute to heterogeneity in cardiovascular disease across the life course. Statins reduce atherosclerotic cardiovascular events in women and men, with no consistent evidence of treatment-effect heterogeneity by sex. In addition to lowering low-density lipoprotein cholesterol, statins influence inflammatory signaling, endothelial nitric oxide bioavailability, oxidative stress, thrombosis, and plaque biology. These effects are biologically relevant to atherosclerosis; however, current clinical evidence does not establish that LDL-independent statin effects confer a uniquely greater benefit in women. Historical pathological studies suggested that plaque erosion may contribute relatively more often to acute coronary thrombosis in selected younger women, whereas contemporary optical coherence tomography studies show similar overall frequencies of plaque rupture and erosion between sexes and important age-related variation. Evidence for a female-specific effect of statins on post-myocardial infarction remodeling, cognition, or pharmacogenomic susceptibility is also insufficient. This narrative review distinguishes established clinical evidence from mechanistic plausibility and unresolved sex-specific hypotheses. It emphasizes equitable implementation of guideline-directed lipid-lowering therapy, careful evaluation of statin-associated symptoms, and the need for adequately powered studies reporting sex-stratified estimates and formal treatment-by-sex interactions.

## 1. Introduction

Cardiovascular disease (CVD) is the leading cause of mortality among women worldwide and remains underrecognized, underdiagnosed, and undertreated. Women share conventional cardiovascular risk factors with men, but the magnitude, timing, and clinical expression of risk may differ across the life course. Hypertensive disorders of pregnancy, gestational diabetes, premature menopause, and polycystic ovary syndrome provide additional opportunities for early risk recognition [1,2].

Inflammation is integral to atherosclerosis, but sex-related differences in immune regulation, hormonal exposure, endothelial function, adiposity, and vascular remodeling may modify inflammatory phenotypes [3,4]. These biological observations should not be conflated with proof that anti-inflammatory or pleiotropic drug effects produce greater clinical benefit in women. A clear distinction is required among three propositions: women and men may differ in disease biology; statins have effects beyond low-density lipoprotein cholesterol (LDL-C) lowering; and those non-LDL effects may or may not have different clinical importance by sex.

Statins remain a cornerstone of primary and secondary prevention of atherosclerotic cardiovascular disease (ASCVD). Large meta-analyses and guideline syntheses support comparable proportional reductions in major vascular events in women and men, with no consistent treatment-by-sex interaction [5,6]. The purpose of this narrative review is therefore not to claim a uniquely female pleiotropic benefit but to summarize established evidence, identify biologically plausible sex-related mechanisms, and define the questions that remain unresolved.

## 2. Cardiovascular Risk and Disease Phenotypes in Women

CVD accounts for approximately one-third of deaths among women globally. Although age-standardized mortality has declined in many regions, ischemic heart disease and stroke remain major causes of death and disability, and concerning trends have been reported among younger women with acute myocardial infarction [1,7,8]. In Brazil, hypertension, obesity, dyslipidemia, hyperglycemia, smoking, and unhealthy dietary patterns contribute substantially to the female cardiovascular burden [2].

Female-specific or female-predominant conditions modify lifetime risk. Hypertensive disorders of pregnancy and gestational diabetes predict later hypertension, diabetes, and CVD; premature menopause is associated with higher long-term cardiovascular risk; and systemic autoimmune diseases, which are more prevalent in women, accelerate vascular inflammation and atherosclerosis [1,2]. Psychosocial stressors, caregiving burden, socioeconomic disadvantage, and depression may further influence prevention, access to care, and adherence.

Sex-specific risk estimates require precise reporting. In INTERHEART, the odds ratio for myocardial infarction associated with hypertension was 2.95 in women and 2.32 in men, whereas the corresponding estimates for diabetes were 4.26 and 2.67 [9]. Current smoking was strongly associated with myocardial infarction in both sexes. A separate meta-analysis reported a female-to-male ratio of relative risks of 1.25 for coronary heart disease associated with current smoking [10]. These measures describe different comparisons and should not be merged into a single “women versus men” odds ratio.

## 3. Sex, Inflammation, and Coronary Pathophysiology

Estrogens modulate endothelial nitric oxide signaling, leukocyte adhesion, cytokine expression, and vascular oxidative stress through receptor-dependent and receptor-independent pathways. Menopause is accompanied by changes in body composition, lipids, insulin sensitivity, and inflammatory markers; however, these changes are heterogeneous and are influenced by age, adiposity, comorbidity, and hormone exposure [3,11]. Accordingly, higher inflammatory biomarker concentrations after menopause cannot be attributed solely to estrogen depletion.

High-sensitivity C-reactive protein (hs-CRP) is the most clinically established inflammatory biomarker in cardiovascular prevention. In long-term follow-up of women, hs-CRP, LDL-C, and lipoprotein(a) each contributed to cardiovascular risk prediction [12]. In JUPITER, rosuvastatin reduced cardiovascular events among individuals with LDL-C below conventional treatment thresholds and hs-CRP ≥ 2 mg/L; women experienced a significant benefit, but the trial did not establish that the benefit in women was mediated predominantly by pleiotropic rather than LDL-lowering effects [13].

Other biomarkers, including interleukin-6, tumor necrosis factor-alpha, and myeloperoxidase, are important research tools but are not supported by assay-independent clinical thresholds for routine sex-specific risk stratification. In particular, a universal myeloperoxidase cut-off should not be proposed because concentrations vary by specimen, assay, pre-analytical handling, population, and endpoint.

### 3.1. Coronary Microvascular Dysfunction and INOCA

Women are disproportionately represented among patients with ischemia or angina and non-obstructive coronary arteries (INOCAs). Coronary microvascular dysfunction and vasomotor disorders may cause ischemia, impaired coronary flow reserve, and persistent symptoms despite the absence of obstructive epicardial disease [14,15]. In the WISE cohort, impaired coronary microvascular reactivity predicted adverse outcomes in women evaluated for suspected ischemia [14].

Inflammation, endothelial dysfunction, autonomic regulation, and structural microvascular remodeling are plausible contributors to INOCA. Nevertheless, evidence that statin pleiotropy produces a greater clinical benefit in women with INOCA than in men is currently insufficient. Trials designed around coronary function, symptoms, and clinical outcomes with prespecified sex analyses are needed.

### 3.2. Plaque Erosion and Rupture

Older autopsy studies suggested that plaque erosion was relatively frequent in selected younger women who died suddenly from coronary thrombosis. These observations generated an important mechanistic hypothesis but arose from highly selected pathological populations.

Contemporary in vivo optical coherence tomography (OCT) studies do not support a simple sex dichotomy in which erosion predominates in women and rupture in men. In a study of 1368 patients with acute coronary syndromes, including 286 women, the overall distribution of culprit plaque rupture and erosion was similar between sexes; the major finding was an age-related increase in plaque rupture and vulnerability features among women [16]. Another OCT analysis also found a similar overall prevalence of erosion in women and men, with age-related patterns within each sex [17]. Thus, plaque erosion should be described as one mechanism of acute coronary syndromes whose prevalence varies with age, smoking, lesion characteristics, and other clinical factors, rather than as the predominant mechanism in women.

## 4. Statin Actions: Established LDL-C Lowering and Biological Effects Beyond LDL-C

Statins inhibit 3-hydroxy-3-methylglutaryl-coenzyme A reductase and reduce hepatic cholesterol synthesis, upregulating LDL receptors and lowering circulating LDL-C. The causal relationship between LDL-C reduction and lower ASCVD risk is supported by genetic, epidemiological, and randomized evidence [5,6].

Inhibition of the mevalonate pathway also reduces isoprenoid intermediates required for prenylation of small GTP-binding proteins such as Rho, Rac, and Ras. Experimental and translational studies show effects on nuclear factor kappa B signaling, endothelial nitric oxide synthase, oxidative stress, leukocyte adhesion, thrombosis, and plaque composition [18,19]. Statins can reduce hs-CRP, as demonstrated in PRINCE and other studies, although the clinical separation of LDL-dependent and LDL-independent effects is difficult [20].

These mechanisms are relevant to atherosclerosis in both sexes. Current evidence supports the statement that statins have biological effects beyond measured LDL-C lowering; it does not establish that these effects are uniquely greater or more clinically important in women. Claims about sex-specific pleiotropic benefit should therefore be presented as hypotheses requiring direct testing.

### 4.1. Inflammation as a Therapeutic Target

CANTOS demonstrated that inhibition of the interleukin-1beta pathway with canakinumab reduced recurrent cardiovascular events independently of lipid-lowering therapy [21]. The primary trial did not provide evidence of a differential treatment benefit associated with higher baseline inflammation in women; this hypothesis would require evaluation through a formal sex-by-treatment-by-inflammation interaction analysis.

COLCOT reported a hazard ratio of 0.77 for its primary composite endpoint after myocardial infarction, whereas LoDoCo2 reported a hazard ratio of 0.69 in chronic coronary disease [22,23]. Women represented approximately 19% and 15% of the respective populations. These trials were not adequately powered to establish efficacy independently in women or equivalence of treatment effects by sex. The absence of a statistically significant interaction is not proof of identical effects.

### 4.2. Post-Myocardial-Infarction Remodeling

Post-infarction remodeling involves inflammation, neurohormonal activation, extracellular matrix turnover, fibrosis, ventricular dilation, and changes in systolic and diastolic function. Experimental and clinical studies suggest that statins may influence inflammatory and fibrotic pathways, endothelial function, and myocardial perfusion [24,25]. High-intensity statin therapy after acute coronary syndromes is clearly indicated to reduce recurrent ASCVD events; however, evidence that statins specifically improve ventricular remodeling through a female-specific mechanism is limited.

Sex-related differences in infarct characteristics and remodeling have been reported, but the literature is heterogeneous and often observational. Few statin studies provide sex-stratified remodeling estimates or formal treatment-by-sex interaction tests. Therefore, a female-specific remodeling benefit should be framed as an unconfirmed mechanistic hypothesis rather than an established clinical effect.

## 5. Clinical Evidence for Statin Therapy in Women

Randomized trials and individual-participant meta-analyses support statin therapy for women according to baseline ASCVD risk and established cardiovascular indications. Proportional reductions in major vascular events are generally similar in women and men, although the absolute benefit depends on baseline risk [5,6]. Underrepresentation of women, particularly younger women and those with pregnancy-related risk factors or INOCA, limits precision for some subgroups.

The central clinical message is therefore equitable implementation of evidence-based lipid-lowering therapies. Women should not receive less intensive prevention because of risk underestimation, atypical symptoms, concerns about adverse effects, or misconceptions regarding protection before menopause. At the same time, mechanistic observations should not be used to imply a superior female-specific response when formal interaction evidence is absent (Table 1).

## 6. Pharmacogenomics, Tolerability, and Alternative Lipid-Lowering Therapy

The SLCO1B1 rs4149056 (c.521T>C) variant reduces OATP1B1 transporter function and is associated most clearly with simvastatin-related myopathy, particularly at high doses [26]. ABCG2 and CYP2C9 variants also influence exposure to selected statins. Current Clinical Pharmacogenetics Implementation Consortium guidance is statin-, genotype-, and dose-specific [27]. Pravastatin and rosuvastatin should not be described as independent of SLCO1B1, because both are OATP1B1 substrates, and rosuvastatin disposition is also influenced by ABCG2.

Female sex and reduced SLCO1B1 function may each be associated with muscle-related adverse effects, but this does not establish a genotype-by-sex interaction. Evidence for such an interaction would require formal evaluation in an appropriately designed study. Observational studies often report more statin-associated muscle symptoms among women, whereas randomized trials show smaller differences and underscore the importance of the nocebo effect, comorbidities, drug interactions, thyroid disease, vitamin D deficiency, frailty, and dose [28].

When symptoms occur, guideline-based management includes confirmation of the temporal relationship, exclusion of secondary causes and interactions, dechallenge and rechallenge when appropriate, use of a different statin or dosing schedule, and addition of non-statin therapy to achieve LDL-C goals. Ezetimibe and monoclonal PCSK9 inhibitors have demonstrated cardiovascular outcome benefit in appropriate populations. Inclisiran produces substantial and durable LDL-C reduction, but a dedicated cardiovascular outcome benefit has not yet been established; ongoing outcome programs are intended to address this question [29].

Bempedoic acid reduced major adverse cardiovascular events in the CLEAR Outcomes trial, which enrolled 13,970 statin-intolerant participants, 6740 of whom were women (48%) [30]. A prespecified sex analysis found no significant heterogeneity in cardiovascular benefit, and a contemporary analysis of the trial cohort reported broadly similar tolerability in women and men [31,32]. Hyperuricemia and gout remain clinically relevant adverse effects.

### Cognitive Function

Randomized trials and systematic reviews have not demonstrated a consistent causal association between statins and clinically important cognitive decline [33]. Subjective cognitive symptoms can occur and should be assessed individually, particularly in older patients with polypharmacy, frailty, or pre-existing cognitive impairment. However, current evidence does not establish a female-specific susceptibility or a clinically validated interaction among statin exposure, APOE genotype, and sex. Current evidence is insufficient to establish female APOE ε4 carriers as a distinctly vulnerable subgroup.

## 7. Evidence Gaps and Research Priorities

Future studies should prespecify sex-stratified analyses, report female enrollment, provide sex-specific effect estimates and confidence intervals, and test treatment-by-sex interactions rather than infer equivalence from nonsignificant subgroup findings. Mechanistic, imaging, biomarker, and clinical outcome evidence should be reported separately.

**Figure 1:**
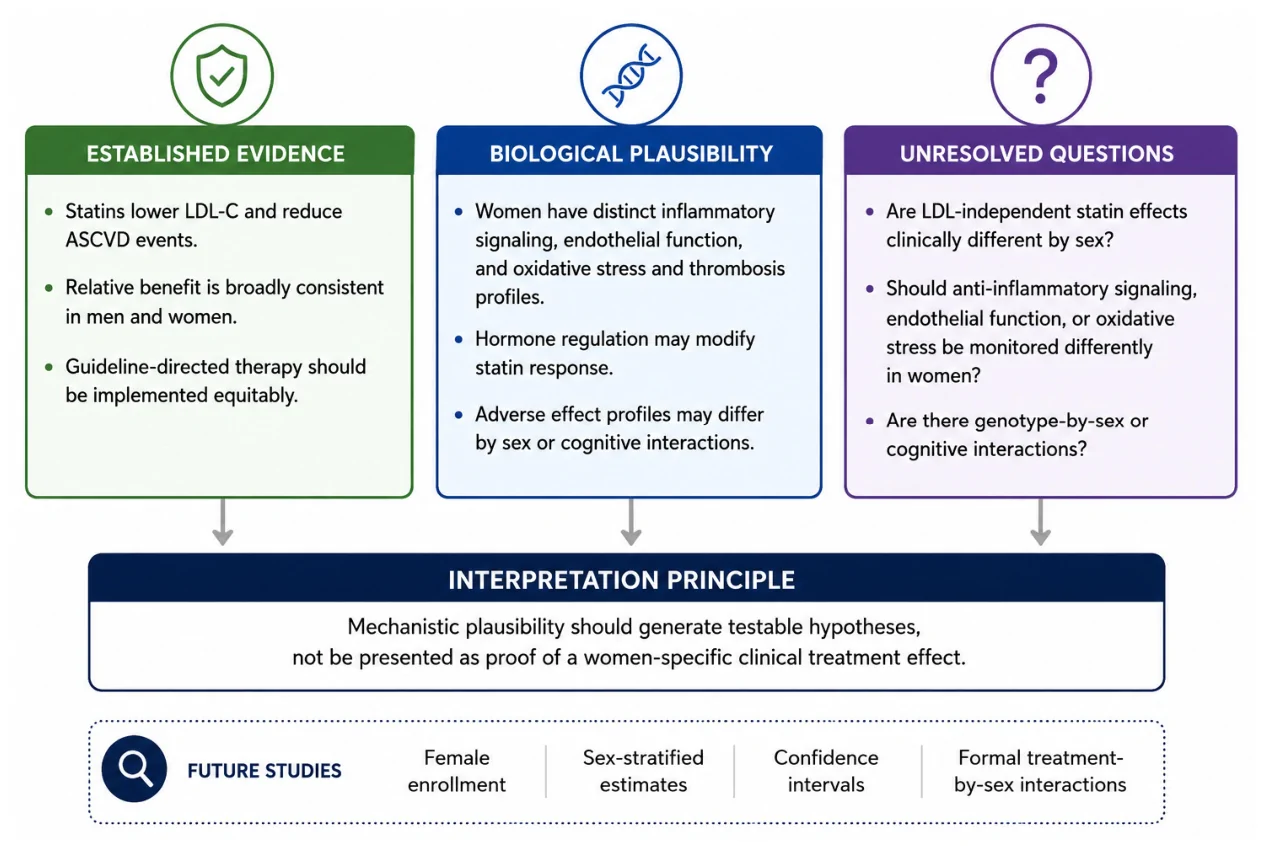
Evidence framework for interpreting statin pleiotropy in women. Established clinical evidence supports statin therapy in women according to ASCVD risk and indications. Biological effects beyond measured LDL-C lowering are plausible and relevant to both sexes, but female-specific clinical importance remains unconfirmed. Future studies should report sex-stratified estimates and formal interaction tests.

Priority areas include women with INOCA and coronary vasomotor disorders, younger women with premature ASCVD, women with prior hypertensive disorders of pregnancy or gestational diabetes, menopause-related transitions in vascular inflammation, and pharmacogenomic studies adequately powered to test genotype-by-sex interactions. Studies of ventricular remodeling should include standardized imaging endpoints and formal sex interaction analyses (Figure 1).

## 8. Conclusions

Statins reduce ASCVD events in women and should be prescribed according to guideline-based risk and clinical indications. Their effects on inflammatory signaling, endothelial function, oxidative stress, thrombosis, and plaque biology provide important mechanistic context, but current evidence does not demonstrate that LDL-independent statin effects confer a uniquely greater clinical benefit in women.

Sex-related differences in cardiovascular biology are real and clinically relevant, yet they must be separated from unproven treatment-effect claims. A balanced interpretation supports equitable lipid-lowering therapy today while recognizing that female-specific pleiotropic, remodeling, cognitive, and pharmacogenomic effects remain important research questions.

## Figures and Tables

**Table 1:** Evidence supporting clinical and mechanistic statements relevant to statin therapy in women.

Clinical Question	Main Evidence Source	Women Represented	Sex-Specific Estimate/Interaction	Certainty	Principal Limitation
Do statins reduce ASCVD events in women?	Individual-participant meta-analysis of randomized statin trials	Large pooled female population	Comparable proportional benefit; no consistent treatment-by-sex heterogeneity	High	Absolute benefit varies with baseline risk; some female subgroups remain underrepresented
Is plaque erosion more common overall in women with ACS?	Contemporary OCT cohorts	286 women in one 1368-patient cohort; additional mixed-sex OCT cohort	Overall rupture/erosion distribution similar; age-related patterns differ	Moderate	Selected patients undergoing OCT; age and clinical phenotype modify prevalence
Are pleiotropic statin effects uniquely more important in women?	Mechanistic and translational studies; clinical outcome trials not designed for this interaction	Variable	No direct proof of greater LDL-independent clinical effect in women	Low/hypothesis-generating	Mechanistic endpoints do not establish sex-specific clinical benefit
Do statins improve post-MI remodeling differently by sex?	General remodeling studies and limited sex-specific observational literature	Often incompletely reported	Formal treatment-by-sex estimates generally unavailable	Low	Few prespecified sex analyses; heterogeneous imaging endpoints
Does SLCO1B1 interact with sex to increase myopathy?	SEARCH pharmacogenetic study; CPIC guideline	Sex interaction not established	Genotype main effect established for selected statins/doses; genotype-by-sex interaction unproven	Moderate for genotype; low for interaction	Statin-, dose-, and ancestry-specific effects
Are cognitive effects different in women?	Randomized trials and systematic reviews	Women included, sex interactions rarely primary	No established female-specific cognitive harm	Moderate	Subjective symptoms and observational confounding; limited formal sex interactions
What is the evidence for bempedoic acid in women?	CLEAR Outcomes and prespecified sex analysis	6740 women (48%)	Cardiovascular benefit without significant heterogeneity by sex	High for overall outcome; moderate for sex subgroup	Subgroup analyses have less precision than overall trial

## Data Availability

No new data were created or analyzed in this study. Data sharing is not applicable to this article.
